# Boron Nitride-Supported Metal Catalysts for the Synthesis and Decomposition of Ammonia and Formic Acid

**DOI:** 10.3390/nano15030212

**Published:** 2025-01-28

**Authors:** Marta Yruela-Garrido, Eduardo Campos-Castellanos, María V. Morales, Inmaculada Rodríguez-Ramos, Antonio Guerrero-Ruiz

**Affiliations:** 1 Instituto de Catálisis y Petroleoquímica, CSIC, 28049 Madrid, Spain; marta.yruela@csic.es (M.Y.-G.); irodriguez@icp.csic.es (I.R.-R.); 2 Departamento de Química Inorgánica y Química Técnica, UNED, Las Rozas, 28232 Madrid, Spain; camposeduardo@ccia.uned.es (E.C.-C.); mvmorales@ccia.uned.es (M.V.M.); 3 Grupo de Diseño y Aplicación de Catalizadores Heterogéneos, Unidad Asociada UNED-CSIC (ICP), UNED, 28049 Madrid, Spain

**Keywords:** boron nitride, metal nanoparticles, supported catalysts, ammonia, formic acid, hydrogen

## Abstract

This review explores the recent advancements in the application of boron nitride (BN) as a support material for metallic nanoparticles, highlighting its potential in fostering sustainable chemical reactions when employed as a heterogeneous catalyst. Two key processes, both critical to hydrogen storage and transport, are examined in detail. First, the reversible synthesis and decomposition of ammonia using BN-supported metallic catalysts has emerged as a promising technology. This approach facilitates the preparation of Ru nanoparticles with precisely structured surface atomic ensembles, such as B5 sites, which are critical for maximizing catalytic efficiency. Second, the review emphasizes the role of BN-supported catalysts in the production of formic acid (FA), a process intrinsically linked to the reuse of carbon dioxide. In this context, hydrogen and carbon dioxide—potentially sourced from atmospheric capture—serve as reactants. BN’s high CO_2_ adsorption capacity makes it an ideal support material for such applications. Moreover, FA can serve as a source of hydrogen through decomposition or as a precursor to alternative chemicals like carbon monoxide (CO) via dehydration, further underscoring its versatility in sustainable catalysis.

## 1. Introduction

The growing demand for clean and sustainable energy sources has driven intense research into new hydrogen storage and production systems, a key energy vector for the decarbonization of the industrial and transportation sectors [1,2,3]. Among the most promising hydrogen storage molecules, ammonia (NH_3_) and formic acid (HCOOH) stand out due to their high hydrogen densities and the possibilities for their synthesis and decomposition under relatively controlled conditions [4,5,6,7,8,9]. However, the efficient production and decomposition of these molecules require advanced catalysts that should be stable, selective, and cost-effective [10,11,12]. Developing these catalysts is crucial for improving sustainable industrial processes, such as the synthesis and decomposition of ammonia and formic acid, given their significant roles in industrial chemistry and future energy storage applications [13,14]. In this context, boron nitride (BN)-supported metal catalysts have emerged as ideal candidates due to the unique physical and chemical properties of this support [10,11,14,15,16,17]. A preliminary and interesting characteristic of this support material is the absence of oxygen atoms in its composition, in contrast to typical inorganics inert metal oxides (SiO_2_, Al_2_O_3_, zeolites, etc.) and reactive metal oxides (CeO_2_, TiO_2_, etc.). This latter group easily gives up oxygen species and produces specific metal–support interactions. But, in the case of inert metal oxides, the participation of oxygen species in some steps of the heterogeneously catalyzed reaction is plausible, particularly when non-noble metallic particles are acting as the catalyst. In short, with BN as a support, it is feasible to determine the intrinsic properties of metallic nanoparticles [17,18], avoiding the participation of oxygen atoms in catalytic reactions where these species are not required.

Boron nitride has proven to be a highly versatile material in the field of heterogeneous catalysis thanks to its distinctive structural and electronic properties [19,20,21,22]. Initially recognized for its hexagonal structure similar to graphite [23], known as hexagonal boron nitride (h-BN), this material exists in various morphologies that significantly expand its range of applications. These include boron nitride nanotubes (BNNTs), boron nitride nanosheets (BNNSs), nanoparticles (NPs), and even more complex forms such as boron nitride haeckelites, each with distinctive characteristics that make them suitable for specific catalytic applications. Boron nitride nanotubes, structurally analogous to carbon nanotubes (CNTs) [24,25,26,27], combine high thermal and chemical stability with unique electrical properties, such as an insulating nature due to a constant bandgap (~5.8 eV) regardless of tube diameter or chirality [19,28]. This makes them ideal supports for metal catalysts in applications requiring high resistance to extreme conditions without compromising the structural stability of the catalyst [29,30,31,32]. On the other hand, boron nitride nanosheets, analogous to graphene [33,34], are ultrathin two-dimensional materials offering a large specific surface area for the dispersion of active metal nanoparticles, favoring their use in catalytic reactions where surface–reactant interactions are critical [19,35,36,37,38,39].

Additionally, boron nitride nanoparticles (BNNPs), which are less studied compared to BNNTs and BNNSs, show potential as catalytic supports due to their high stability and ease of functionalization [11,40,41]. These particles can be synthesized with precise control over their size and composition, enabling the tuning of their electronic and chemical properties for specific applications in heterogeneous catalysis [42,43]. Likewise, BN nanofibers, elongated structures with nanometric diameters, combine the high thermal stability of the material with a morphology that facilitates the transport of reactants and products through the catalytic matrix [14,44,45]. It is worth mentioning a less conventional but intriguing form: boron nitride haeckelites, which exhibit distorted atomic networks with pentagons and heptagons in addition to traditional hexagons [46]. These defects alter the material’s electronic and mechanical properties, increasing its adsorption capacity and potentially creating new active catalytic sites [47,48,49].

From a surface perspective, BN is known for its chemical inertness in its pure state [10,50,51]. However, this characteristic also makes it a highly selective and stable support, avoiding unwanted interactions with reactants or reaction products [36,52]. For example, regarding H_2_ adsorption, the van der Waals interactions between the BN surface and H_2_ molecules are relatively weak, resulting in low storage capacities under ambient conditions [22,53]. This behavior is advantageous in applications where the catalytic support should not excessively adsorb H_2_, facilitating its release in dehydrogenation reactions, such as formic acid decomposition [54,55]. However, to enhance efficiency in processes where H₂ activation is crucial, BN can be functionalized through doping with transition metal atoms, or by introducing structural defects such as nitrogen or boron vacancies [29,49,53,56,57,58,59,60,61,62,63,64]. These modifications generate active sites that promote the chemisorption of H_2_, enabling more efficient activation of the molecule for participation in catalytic reactions [41,65,66,67].

CO_2_, on the other hand, due to its polarizable nature, exhibits specific behavior in its interaction with active surfaces. In its pure state, BN has a limited CO_2_ adsorption capacity due to the lack of active functional groups that could favor strong chemical interactions [68,69]. Nevertheless, functionalizing its surface with groups like hydroxyls (-OH) or amines (-NH_2_), or introducing structural defects, significantly improves its adsorption capacity [70,71]. These modifications increase the density of active sites and strengthen the chemical interactions between CO_2_ molecules and the support. The chemisorption of CO_2_ on modified boron nitride surfaces has been confirmed in both theoretical and experimental studies [72,73]. This behavior is especially relevant in reactions such as formic acid synthesis from CO_2_, where efficient activation of the molecule is a critical step [74,75,76]. Moreover, porous BN structures, such as fibers and nanosheets, offer a high surface area and a high proportion of micropores, maximizing their adsorption capacity [69,71]. For instance, “3D functionalized flower-like boron nitride nanosheets” (FBNNSs) have demonstrated adsorption capacities of up to 74.4 mg CO_2_/g, thanks to the combination of physical and chemical interactions on their surface. These properties position BN as a promising material in sustainable carbon capture technologies. These unique surface properties also help prevent the sintering of supported metal nanoparticles, a common issue in heterogeneous catalysis that occurs when metal particles agglomerate at high temperatures, reducing the available surface area for reactions [12,42,77,78]. BN, due to its chemical stable surface and its ability to maintain high nanoparticle dispersion, is particularly effective in mitigating this phenomenon [50,72,79].

Heterogeneous catalysis presents specific challenges and opportunities when employing BN as a support, especially in high-temperature systems. Its remarkable stability and chemical resistance provide significant advantages over other conventional supports such as metal oxides or activated carbons [50,80,81]. Furthermore, BN’s ability to withstand thermal degradation and maintain structural integrity under reactive conditions makes it a promising alternative for highly demanding catalytic applications [82,83,84]. In contrast with homogeneous catalysts, which often require complex separation processes, heterogeneous catalysts can be easily separated and recycled, making them more practical and sustainable for large-scale operations [85].

**Scheme 1:**
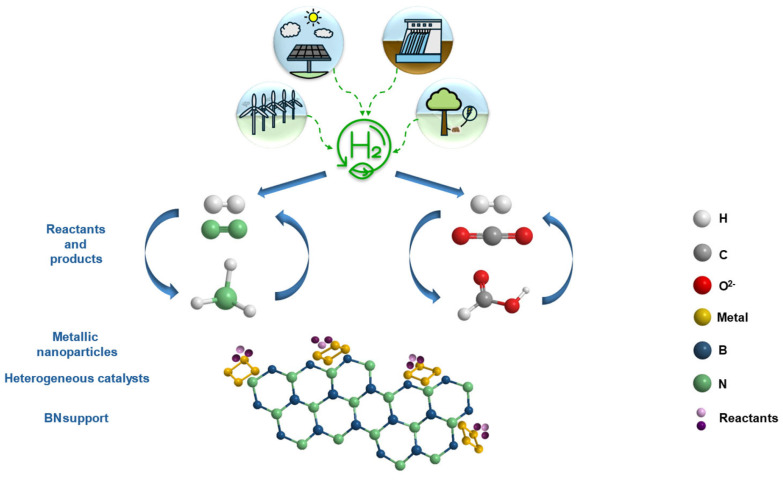
Conceptual representation of the highlighted developments of new heterogeneous catalysts and related technical processes compiled in this review.

This review highlights the use of boron nitride as a support for metal catalysts in heterogeneous catalysis, examining both the opportunities and challenges associated with its use in reactions such as the synthesis and decomposition of ammonia and formic acid. It delves into the structural and electronic properties of BN that enhance its performance in these systems, along with strategies for its modification and optimization for catalytic purposes. Scheme 1 provides a comprehensive overview of the processes and catalytic materials discussed in this review.

## 2. Catalytic Application of Boron Nitride-Supported Metal Nanoparticles

### 2.1. Ammonia Synthesis

Ammonia synthesis remains one of the most important industrial reactions, primarily used for fertilizer production. Since the implementation of the Haber–Bosch process over a century ago, the development of more efficient catalysts has remained a central objective to minimize energy consumption and improve process sustainability [86,87]. In the field of ammonia synthesis, three generations of catalysts can be identified: (i) first-generation catalysts: iron-based systems promoted with alumina, potassium oxides, calcium oxides, silica, and barium oxide [88,89,90]; (ii) second generation catalysts: ruthenium-based catalysts supported over activated carbon and promoted with K_2_O, BaO, or Cs_2_O [91,92]; and (iii) third-generation catalysts: advanced systems utilizing metals like ruthenium, copper, or nickel, supported on more innovative materials such as nitrogen-doped carbon nanotubes [93], magnesium oxide [94], or boron nitride, with promoters like barium or cesium oxides [95,96]. This review focuses on the latest generation of catalysts, particularly those supported on boron nitride. These catalysts have garnered attention as a promising alternative to traditional iron-based systems, owing to their ability to operate under milder pressure and temperature conditions, marking a significant step toward more sustainable ammonia synthesis.

Boron nitride, especially in its hexagonal form, has proven to be an exceptional support in this field. Unlike other supports such as graphitized carbon, which can deactivate due to methanation reactions [97], BN remains inert and stable at high temperatures and pressures, ensuring a longer catalyst lifespan [98]. Moreover, h-BN minimizes metal–support interactions, enabling a high dispersion of metal particles and maximizing the active sites on the catalyst surface [25].

#### 2.1.1. Effect of Barium Doping on Ru/BN Catalysts

Barium doping in Ru/BN catalysts has been identified as a key strategy for enhancing catalytic activity. Barium acts as both an electronic and structural promoter [99]. Electronically, Ba modifies the charge density on ruthenium, facilitating the activation of nitrogen molecules, which is the rate-limiting steps in the ammonia synthesis reaction. Structurally, Ba stabilizes ruthenium particles and prevents sintering, even after prolonged operation cycles [100,101].

Jacobsen [102] compared two ruthenium catalysts: one unpromoted (Ru/BN) and another barium-promoted on hexagonal boron nitride (81 m^2^/g) (Ba-Ru/BN), with 4.5 wt% Ru/BN and 5.6% Ba contents. The barium-promoted catalyst showed a catalytic activity exceeding that of the unpromoted catalyst by more than two orders of magnitude, achieving an activity of 2981 mL·g^−1^ h^−1^. Additionally, the stability results of Ba-Ru/BN during 3500 h of operation without signs of deactivation were attributed to (1) the thermodynamic and structural stability of BN under the test conditions, which ensured the optimal dispersion of ruthenium particles (2.0–2.5 nm) while minimizing metal–support interactions, keeping active sites available for the reaction, and (2) the influence of the BN support on the morphology of ruthenium crystals, as suggested by the activity observed in barium-promoted ruthenium catalysts [103]. Building upon Jacobsen’s findings, Szmigiel et al. [104] synthesized barium-promoted ruthenium catalysts supported on boron nitride, using two types of commercial BN: BNS (190 m^2^/g) and HCV (40 m^2^/g). These supports were modified by heating them under an ammonia flow at high temperatures (700–800 °C), which increased their surface area and reduced boron oxide (B_2_O_3_) impurities, known to act as deactivating agents. Ba-Ru/BN catalysts with 9 wt% Ru and 4.3 wt% Ru contents and various barium-to-ruthenium molar ratios were activated under a high-pressure ammonia-rich flow (90 bar, 10% NH_3_ in H_2_:N_2_ = 3:1), which proved to be more effective than conventional H_2_/N_2_ activation. This procedure rapidly removed B_2_O_3_ impurities and significantly improved catalytic activity, increasing it by 2–3 times compared to unmodified BN supports. The most active catalyst was supported on HCV with 4.3 wt% Ru and a Ba:Ru molar ratio of 1:1, achieving an activity of 1.3 gNH_3_/g_Ru+BN_·h, aligning with the observed trend that catalysts with a lower barium content are generally more active than those with a higher barium content (Ba:Ru = 3:1). However, these results are still lower than those achieved with carbon-based supports [105,106] and those reported by Jacobsen et al. [102], suggesting that B_2_O_3_ might still act as a deactivating agent, offsetting the promotional effect of barium. This underscores the importance of developing high-purity BN supports with a minimal oxygen content to attain activity levels comparable to those of carbon-based catalysts.

The evaluation of BN supports synthesized via temperature-programmed nitridation (BN-1) and reduction (BN-2), along with a commercial BN (BN-3), in barium-promoted ruthenium catalysts (Ru-Ba/BN), underscores the importance of optimizing BN synthesis conditions and purity [77]. The BN supports exhibited hexagonal structures with surface areas of 103 m^2^/g and 138 m^2^/g for BN-1 and BN-2, respectively, and 22 m^2^/g for BN-3. N_2_ adsorption–desorption isotherms revealed a mesoporous behavior for BN-1, while BN-2 showed a more uniform but smaller pore size distribution. Of the three BN-based catalysts, only Ru-Ba/BN-1 exhibited a rather promising activity, achieving an ammonia concentration of 7.3% under the conditions of 475 °C, 10 MPa, and 10,000 h^−1^, while maintaining stable activity after treatments at 550 °C for 30 h. The Ru-Ba/BN-2 and Ru-Ba/BN-3 catalysts exhibited much lower activities (1% and 0.7% ammonia concentration, respectively), likely due to the limited porosity of BN-2 and the low surface area of BN-3, hindering Ru dispersion in both cases. Despite the lower ammonia conversion (7.3%) compared to carbon-supported catalysts [106,107,108], this study highlighted the superior thermal stability of Ru-Ba/BN catalysts, even after prolonged treatment at 550 °C [77].

Yang et al. [109] investigated a composite support consisting of magnesium oxide and hexagonal boron nitride to further advance the development of innovative supports for ruthenium-based ammonia synthesis catalysts. They explored a composite support made of magnesium oxide and hexagonal boron nitride. This support combines the unique properties of both materials: the structural and thermal stability of h-BN with the high basicity of MgO, which increases the electronic density of ruthenium active sites. The catalysts obtained, Ba-Ru/MgO/h-BN, containing 5 wt% Ru and a Ba to Ru molar ratio of 1.0, demonstrated that the mass ratio of MgO to h-BN influences catalytic activity, which increased with higher MgO contents. The catalytic activity measured at 450 °C, 5 MPa, and 5000 h^−1^ for Ba-Ru/MgO/h-BN [8:2], Ba-Ru/MgO/h-BN [6:4], and Ba-Ru/MgO/h-BN [5:5] was 472.5, 467.6, and 429.4 mL/gcat·h, respectively, significantly outperforming Ba-Ru/MgO. X-ray diffraction (XRD) and high-resolution transmission electron microscopy (HRTEM) studies confirmed that composite-supported catalysts featured small, well-dispersed Ru particles, with smaller average sizes than those in systems supported exclusively on h-BN or MgO. By integrating these components in controlled proportions, the catalysts achieved improved Ru dispersion and introduced additional basic sites that increased the electronic density of active sites, optimizing the dissociation of molecular nitrogen (N_2_) and enhancing the catalytic activity in ammonia synthesis. This approach represents a significant step toward more efficient and versatile supports for industrial applications.

The size of ruthenium particles is a key parameter for maximizing catalytic activity in ammonia synthesis, and different studies have documented optimal sizes associated with specific supports. The catalysts reported by Jacobsen et al. [102] presented ruthenium particles with an average size of 2.0–2.5 nm. This range ensures a high density of B5-type active sites [103]. Following this work, Hansen et al. [110] delved deeper into the role of barium as a promoter in Ba-Ru/BN catalysts, employing in situ transmission electron microscopy to show how small ruthenium particles (<1 nm) on h-BN initially suffer sintering to reach optimal sizes of around 2 nm during activation. This confirms the structural sensitivity of the ammonia synthesis reaction. Two main barium structures were identified: (1) an amorphous phase partially covering ruthenium crystals and (2) a well-dispersed structure of barium oxide on specific ruthenium sites. The results suggest that Ba atoms, distributed as individual atoms near the edges of the crystals (B5 sites) and bound to oxygen, are responsible for the electronic promotion of the catalyst by increasing the electron density at the B5 sites of ruthenium, which are critical for the adsorption and cleavage of nitrogen molecules. This effect results in a significant increase in catalytic activity, with improvements of up to two orders of magnitude in ammonia conversion compared to unpromoted catalysts. Furthermore, barium was observed to be highly mobile under reaction conditions, maintaining constant coverage of the active sites. This behavior ensures catalyst stability and reinforces its effectiveness during prolonged operations.

Similarly, Szmigiel et al. [104] reported average particle sizes of 6–7 nm in Ba-Ru/BN catalysts, with variations depending on the support used (BNS vs. HCV) and the thermal treatments applied. However, the presence of impurities such as B_2_O_3_ in the support can negatively impact catalytic performance. B_2_O_3_ is found both on the surface of BN and on the metal particles under ammonia synthesis conditions, forming a composite layer of barium and oxygen along with B_2_O_3_ species that interact with barium oxide, partially covering the ruthenium particles. This coating not only reduces the number of available active sites but also nullifies the promotional effect of barium, thereby limiting the catalyst’s efficiency. This phenomenon highlights the critical relationship between ruthenium particle size and catalytic activity: while particle sizes of 2–5 nm maximize the density of B5-type active sites, larger sizes, such as the 6–7 nm observed by Szmigiel et al. [104], may be less effective if partially covered by impurities or undesired reactive layers.

The catalysts synthesized by Xu et al. [77] clearly demonstrated a relationship between particle size and conversion. For the Ru-Ba/BN-1 system, an average particle size of 33.1 nm was associated with an ammonia conversion of 7.3% (see in Table 1) under the conditions of 475 °C and 10 MPa, making it the most efficient among the BN supports used, due to a relatively higher dispersion (4.07%). In contrast, for Ru-Ba/BN-2 and Ru-Ba/BN-3, where the ruthenium particles reached sizes of 77.5 nm and 88.8 nm, respectively, the catalytic activity decreased drastically, achieving conversions of only 1.0% and 0.7%. This behavior was attributed to the lower dispersion of ruthenium and reduced access of reactants to active sites due to excessive particle growth. By comparison, with a Ru-Ba/AC catalyst, which exhibited significantly smaller metal particle sizes (8.4 nm), a superior ammonia conversion of 12.8% was achieved. This underscores the importance of controlling both the particle size and support characteristics to maximize catalytic activity in ammonia synthesis. These results highlight that intermediate particle sizes, along with supports that promote high dispersion, are ideal for optimizing the efficiency of ruthenium catalysts.

On the other hand, using MgO/h-BN-supported catalysts, Yang et al. [109] revealed through HRTEM analyses that the MgO/h-BN [8:2] support allowed for significantly smaller and more equidimensional ruthenium particles compared to other MgO/h-BN ratios. This reduced particle size correlated with a higher catalytic activity, achieving a production rate of 506.9 mL/gcat·h at 425 °C and 5.0 MPa. In contrast, supports with higher h-BN proportions (such as MgO/h-BN [2:8]) showed a less uniform particle size distribution, potentially reducing the catalytic activity. The h-BN structure plays a crucial role by inducing epitaxial growth of flat ruthenium particles, a feature shared with materials like graphitic carbon [98], where strong in-plane atomic bonding and weak interlayer interactions favor the orderly growth of metal particles. This controlled growth optimizes the formation of active sites, such as B5 sites [111]. In particular, this behavior is observed in balanced proportions, where MgO provides additional basicity and enhances ruthenium dispersion, achieving a higher active site density and significantly improving catalytic activity in ammonia synthesis. Studies on Ru/MgAl_2_O_4_ showed that catalytic activity can decrease if the particles are too small (<1 nm), due to a low availability of B5 sites, while larger sizes (>5 nm) reduce the active surface area [103]. This highlights the importance of optimizing the particle size and support characteristics for effective catalyst performance. The key conclusions of these studies are that the optimal ruthenium particle size for ammonia synthesis generally ranges between 2 and 4 nm, depending on the support and its properties. This control is influenced by factors such as support-induced morphology, thermal treatments, and impurity removal, highlighting the importance of advanced support designs to maximize catalytic activity [103,112].

**Figure 1:**
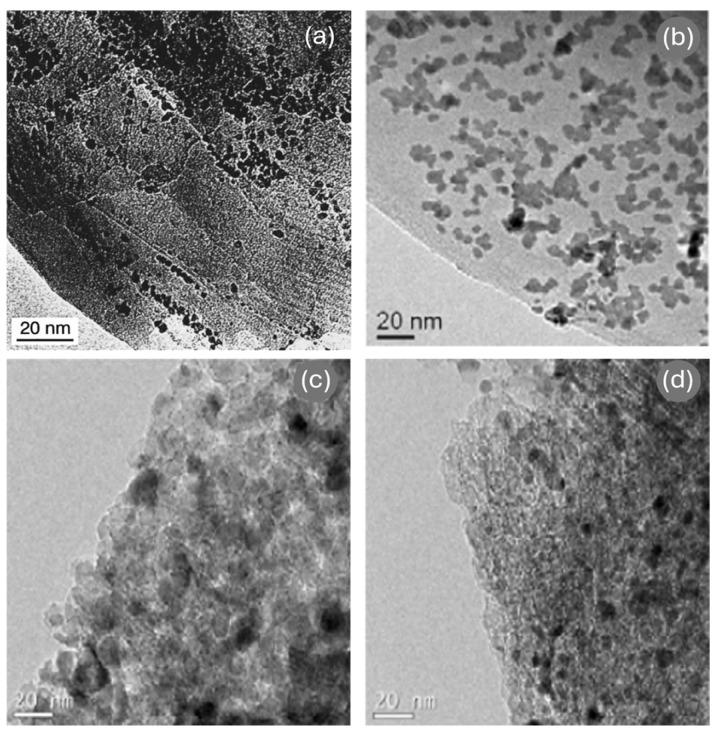
TEM images of (**a**) Ba-Ru/BN catalyst after 1000 h of operation at 550 °C. (**b**) Ru-h-BN catalyst after reduction due to exposure to H_2_ at 650 °C for 6 h. (**c**) Ba-Ru/MgO/h-BN [8:2] BN catalyst after reduction due to exposure to H_2_ at 650 °C for 6 h. (**d**) Ba-Ru/MgO/h-BN [5:5] BN catalyst after reduction due to exposure to H_2_ at 650 °C for 6 h. Reprinted from [102] and [109] with permission from Elsevier, copyrights 2001 and 2012 respectively.

The transmission electron microscopy (TEM) images in Figure 1 show that the particle size of ruthenium in (a) the Ba-Ru/BN catalyst is the smallest, primarily located along the steps on the basal planes of the boron nitride support. Additionally, in the catalysts synthesized by Yang et al. [109], the Ru/h-BN (b) presents irregular and flat particles, while those supported on MgO/h-BN (c and d) display round Ru particles with smaller average sizes compared to Ru/h-BN.

#### 2.1.2. Comparison of Boron Nitride-Supported Catalysts with Other Supports

Although Ru-based catalysts supported on graphitized carbon have been successfully employed in industrial applications, they exhibit significant disadvantages, such as support methanation under high hydrogen pressures, which limits their lifespan. In contrast, BN does not undergo degradation under these conditions and has proven to be a more stable and efficient support, especially when combined with promoters like Ba. Table 1 shows a comparison of the catalytic activities achieved with different supports and promoters. The results presented in this table demonstrate that BN supports of Ru nanoparticles offer an excellent alternative to MgO- or carbon-based material supports in terms of specific catalytic activities in the ammonia synthesis reaction. Furthermore, BN must be superior in durability when acting as a catalyst in reaction due to the high thermal and chemical stability of BN materials. In terms of the promoting effect of Cs in the Ru catalysts supported on C or MCP [105,107], it should be mentioned that the cesium species (Cs^0^) act as an electronic promotor of the Ru nanoparticles.

**Table 1:** Catalytic activity in ammonia synthesis of boron nitride-supported catalysts and other supports. These are abbreviated as boron nitride materials: BN and HCV; active carbons: C or AC; mesoporous carbon plates: MCP; and multiwall carbon nanotubes: MWNTs.

Ref.	Catalyst	Molar Ratio	wt% Ru	T (°C)	Catalytic Activity (ml·g^−1^·h^−1^)
[102]	Ba-Ru/BN	Ba/Ru: 1:1	4.5	400	2981
[104]	Ba1-Ru4.3/HCV_NH3_	Ba/Ru: 1:1	4.3	400	1865
[77]	Ru-Ba/BN	Ba/Ru: 1:1	4	475	2061
[109]	Ba-Ru/MgO/h-BN [8:2]	Ba/Ru: 1:1	5	450	473
[105]	Ba-Ru9.1/C	-	9.1	400	4959
[105]	Ba-Cs-Ru9.1/C	-	9.1	400	9835
[105]	Ba-Cs-Ru23.1/C	-	23.1	400	17,960
[108]	Ba-(Ru/AC)	Ba/Ru: 1	4.3	400	929
[92]	Ru/MgO	-	3.4	315	532
[107]	Cs-Ru/MCP	Cs/Ru: 2.5	10	370	358
[107]	Cs-Ru/MCP	Cs/Ru: 2.5	10	410	1047
[113]	K/Ru/MWNTs	K/Ru: 1:3	-	420	37

### 2.2. Ammonia Decomposition

Ammonia decomposition is positioned as an essential technology in the hydrogen economy, enabling the production of clean hydrogen, free from carbon contaminants (COx), from a molecule with a high energy storage density that is easy to transport. This endothermic process follows a multistep pathway that includes the adsorption of NH_3_ on the catalyst’s active surface, N-H bond cleavage, H_2_ formation, and N_2_ desorption. Among these steps, molecular nitrogen desorption is generally the most limiting step due to the high bond energy required, ranging from 450 to 650 kJ/mol [114]. A key parameter in designing efficient catalysts for low-temperature ammonia decomposition is the metal–nitrogen bond energy. While ammonia must be adsorbed on the active metal for activation, an excess of strongly adsorbed nitrogen atoms can poison the metal’s active sites. Therefore, there is an optimal nitrogen binding energy for these catalysts, within the range of 544–586 kJ/mol, with the maximum activity observed at 561 kJ/mol, which is lower than the binding energy required for ammonia synthesis [115].

The development of advanced catalysts with high activity, stability, and selectivity is crucial to optimizing this process and overcoming the associated kinetic barriers. Transition metal-based catalysts [115], such as those based on cobalt or nickel, supported on basic oxides like MgO or Al_2_O_3_ [6,96,116], offer acceptable activity but require extremely high temperatures (>700 °C) in order to achieve efficient conversion due to the limited metal–support interactions and sintering of metal nanoparticles [117]. Ruthenium-based catalysts, supported on alumina, carbon nanotubes [95,118], or activated carbon [119,120], represent a significant advancement by reducing operating temperatures [121] and improving the process efficiency due to the high intrinsic activity of ruthenium. However, the high cost of this noble metal limits its large-scale implementation.

**Figure 2:**
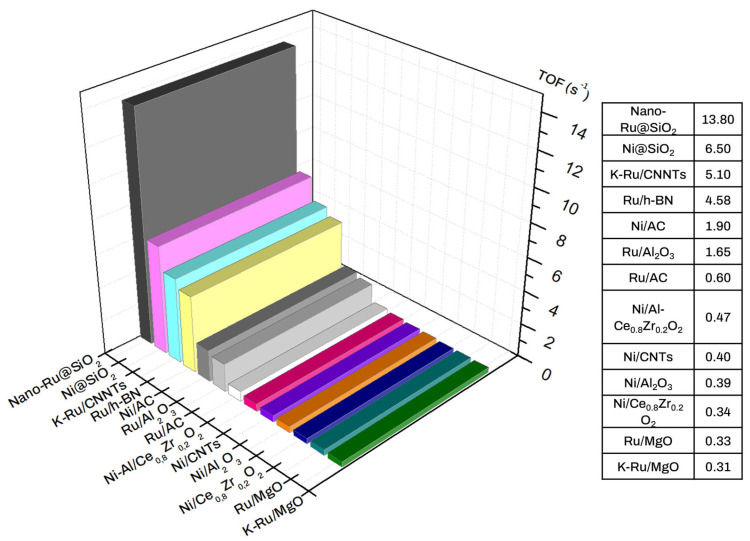
Turnover frequency (TOF) comparison of Ru and Ni catalysts supported on various materials for ammonia decomposition [114,126,127,128,129,130,131,132].

Currently, catalyst design has evolved towards advanced supports like boron nitride and hybrid systems, where BN not only acts as a thermally stable support but also actively contributes to improving nanoparticle dispersion, minimizing sintering, and optimizing electronic transfer to active sites [122]. These advancements have enabled nickel- and ruthenium-based catalysts supported on BN to achieve high conversion rates at lower temperatures, which contribute to sustainability and industrial scalability. Figure 2 presents a comparison of the turnover frequencies (TOFs) of different catalysts based on Ru and Ni. Today’s commercially utilized ruthenium-based catalysts supported on carbon are among the most efficient, achieving ammonia conversion values exceeding 90% at temperatures around 673 K, with a hydrogen production rate of up to 1788 mmol/gcat·h [123]. The use of support materials like activated carbon and carbon nanotubes enhances ruthenium particle dispersion, inhibits the metallic particle growth and increases catalyst stability at temperatures up to 773 K, which arethe main problems of deactivation [124]. However, high ruthenium costs have prompted the exploration of alternatives using more abundant metals like nickel, which, when combined with advanced supports such as BN or CeO_2_, also demonstrates high activity and stability [125]. Figure 2 illustrates that BN is a highly suitable support for Ru nanoparticles, making it a promising option to be applied as heterogeneous catalyst in the ammonia decomposition reaction.

The performance of catalysts in ammonia decomposition depends not only on the active phase used but also on the support and promoters. In general, catalytic activity improves with high-surface-area supports, which enhance dispersion and reduce the particle size of the metal, and with electron-donating promoters such as K, Cs, or Ba [96]. The supports can stabilize the size and morphology of metal particles and increase the exposure of their active sites, while influencing the electronic structure of the supported metal nanoparticles. Catalyst basicity plays a crucial role in enhancing catalytic activity and strengthening the ability to form bonds with nitrogen atoms, thereby improving the efficiency of NH_3_ decomposition [96,123]. Additionally, temperature and concentration influence the kinetic energy of the molecules, potentially enhancing the reaction rate, although excessive temperatures can damage the catalyst [133].

**Figure 3:**
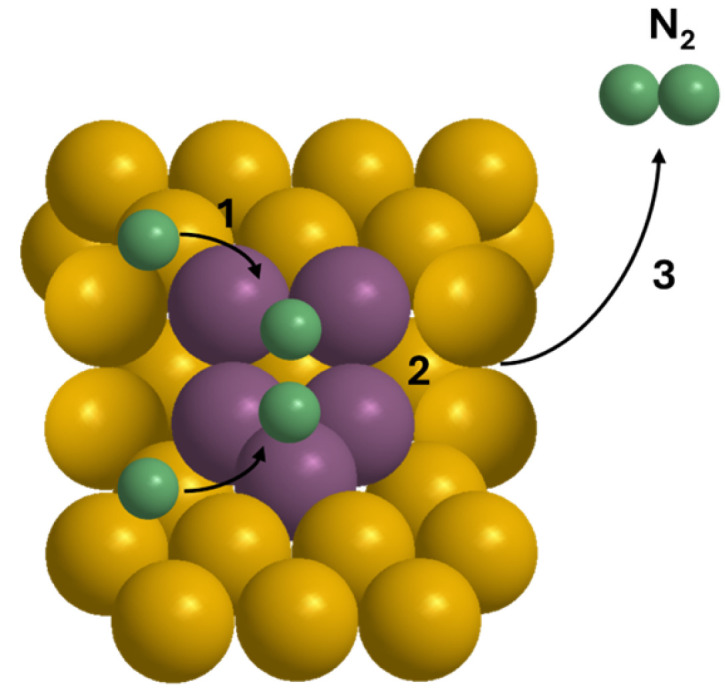
Schematic representation of B5 surface sites exposed at the surface of Ru nanoparticles, which are involved in the associative adsorption of the two N atoms to form N_2_ molecules. The proposed stages of this rate-determining step in the ammonia decomposition reaction are (1) N atoms diffusion to the B5-type site, (2) association of N atoms to form a N_2_ molecule, and (3) desorption of the N_2_ molecule. Image and explanations taken from reference [134]. Reprinted from [134] with permission from Springer Nature, copyright 2009.

An important approach involves the use of ruthenium catalysts supported on BN (Ru/BN), where the hexagonal morphology of the support guides the heteroepitaxial growth of ruthenium nanoparticles rich in B5 sites. These sites are recognized as the most active for NH_3_ decomposition due to their low activation energy for N-H bond cleavage and N_2_ desorption [92]. A recent study [114] developed ruthenium nanoparticle-based catalysts rich in B5-type sites supported on hexagonal boron nitride, which showed exceptional performance in ammonia decomposition. Using a heteroepitaxial growth approach, the interaction between Ru nanoparticles and the h-BN support was optimized, maximizing the exposure of B5 active sites (see Figure 3). These sites are critical for nitrogen molecular desorption, the limiting step in ammonia decomposition, due to the high bond energy required.

**Figure 4:**
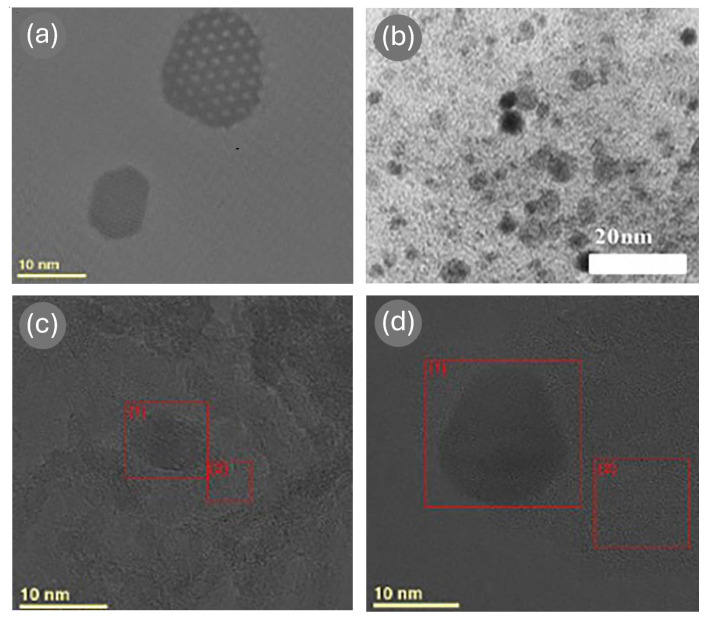
TEM images of (**a**) the Ru nanoparticles supported on the h-BN sheets; (**b**) RuAC-2 catalyst reduced at 973 K; (**c**) Ru/Al_2_O_3_ catalyst4]; (**d**) Ru/SiO_2_ catalyst. Reprinted from [114] with permission from John Wiley and Sons, copyright 2022 and from [134] with permission from Springer Nature, copyright 2009.

The stability of Ru nanoparticles supported on h-BN is notably higher than that on Al_2_O_3_ and SiO_2_, which is attributed to the BN overlayers that formed during the preactivation process. These layers partially cover the Ru nanoparticles, preventing agglomeration and stabilizing their average size at approximately 8.6 nm, even after 80 h of reaction [114]. This protective effect reduces the system’s surface energy while maintaining the morphology and density of active sites, which are essential for catalyst efficiency during ammonia decomposition. The TEM images presented in Figure 4 show that Ru nanoparticles supported on h-BN (a) exhibit a uniform and stable structure, while particles supported on Al_2_O_3_ (c) and SiO_2_ (d) show significant size increases, reaching up to 25.7 nm for SiO_2_, indicating the absence of effective stabilization mechanisms like those provided by h-BN. This behavior underscores the importance of h-BN as not only a chemically inert support but also an active element in preserving the structure and functionality of the catalyst under prolonged operating conditions. Additionally, the support interacts electronically with Ru, increasing the electronic density at active sites and weakening Ru-N bonds, facilitating ammonia decomposition at lower temperatures [135]. The catalytic activity results were also remarkable. At 450 °C, the Ru/h-BN catalyst achieved hydrogen production rates of up to 1133 mmol H_2_/gcat·h, significantly outperforming conventional catalysts supported on alumina or silica. The system also demonstrated high operational stability, maintaining activity over extended periods without evidence of sintering, ensuring a prolonged lifespan under continuous reaction conditions. The Ru/h-BN catalyst, activated for 12 h, also exhibited a promising turnover frequency of 16,498 h^−1^, among the highest recorded under similar conditions (450 °C and GHSV of 60,000 mL NH_3_/gcat·h) (see Figure 2). Its specific activity, measured as the TOF per active site, reached 10,748 h^−1^, outperforming previously reported catalysts by a factor ranging from 3.8 to 54.5 [114]. These results establish Ru/h-BN as one of the most effective catalysts for ammonia decomposition at low temperatures and high flow rates, making it a crucial candidate for industrial-scale applications. Moreover, two additional aspects about this Ru/h-BN catalyst should be noted: the very low loading of Ru (1 wt%) required to prepare this extra-active catalyst, the excellent stability during the reaction (that lasted more than 80 h), and the stabilized operation with ammonia conversion levels near 30%.

Table 2 presents the specific catalytic activities of Ru nanoparticles supported on different materials under various reaction conditions. The catalyst K-Ru/MgO-CNTs achieved the highest hydrogen production rate.

To address the limitations posed by the high cost of ruthenium, Zhou et al. [137] introduced an innovative approach to improving nickel-based catalysts for ammonia decomposition using hybrid CeO_2_-BN supports. This design aimed to tackle the typical challenges of conventional catalysts, such as the agglomeration of Ni nanoparticles and limited metal–support interactions. The hybrid CeO_2_-BN support enhances these interactions through the spatial confinement provided by BN, stabilizing Ni nanoparticles by maintaining their reduced size (~4.4 nm), significantly improving their dispersion (2.4%) compared to the pure Ni/ CeO_2_ system (1.4%) and increasing the specific surface area. High-angle annular dark-field scanning transmission electron microscopy (HAADF-STEM) images revealed a strong overlap in the positions of Ni, Ce, and O at the BN active edge sites and basal planes, maximizing the interaction between these elements. This synergy between BN and CeO_2_ not only minimizes agglomeration but also enhances the interaction between Ni and the support, creating oxygen vacancies and increasing the proportion of Ce³⁺ in cerium oxide. Furthermore, the electronic enrichment of Ni nanoparticles facilitates ammonia activation and promotes molecular nitrogen desorption, overcoming critical kinetic barriers in NH_3_ decomposition [138]. The experimental results showed that the Ni/CeO_2_-BN catalyst achieved 90% NH_3_ conversion at 600 °C, with a hydrogen production rate of 1788 mmol H_2_/g_cat_·h. This performance significantly surpasses that of catalysts based solely on CeO_2_ or BN, highlighting the synergistic effect of the hybrid support. Complementary spectroscopic techniques (XPS, Raman, and DRIFTS) and a temperature-programmed reduction (TPR) analysis confirmed that the interaction between Ni and the CeO_2_-BN support increases Ni’s affinity for NH_3_, lowers the NH_3_ adsorption energy, and facilitates the recombination of nitrogen atoms on the catalyst surface to form N_2_. Additionally, stability studies have indicated that the catalyst can maintain its activity for over 50 h of continuous operation, positioning it as a promising solution for sustainable industrial hydrogen production. With this work, Zhou et al. [137] introduce a generalizable strategy for enhancing catalysts through hybrid support design, offering an effective and scalable approach to optimizing ammonia conversion in hydrogen generation systems that is free from COx emissions.

Table 3 collects the specific catalytic activities obtained with Ni nanoparticles supported on varied materials under various reaction conditions. In summary, while the Ni nanoparticles supported on CeO_2_-BN hybrid materials exhibited high catalytic hydrogen production rates, other materials, such as Ce_0.8_Zr_0.2_O_2_, demonstrated significantly higher catalytic performances.

The role of BN supports in sustainable hydrogen applications, but not directly in isothermal catalytic reactions, has recently been described. Badakhsh et al. [1], leveraging the properties of boron nitride to enhance thermal and chemical stability, explored an autothermal recirculating reactor (ARR) system designed for ammonia decomposition in portable and sustainable hydrogen generation applications [116,141]. This approach addresses the high-temperature challenges and endothermic nature of the reaction by using an innovative design that combines hydrogen combustion as a carbon-free heat source with boron nitride coatings. The proposed system, employing a Ru/La-Al_2_O_3_ catalyst, includes a double-tube reactor where the inner wall material, copper coated with BN (Cu-BN), serves as a heat transfer medium between the combustion chamber and the catalytic bed. The BN ceramic coating, used as a refractory agent, protects the copper against thermal and chemical degradation, enabling a more uniform thermal distribution. This minimizes energy losses and increases reactor durability, making it suitable for temperature-sensitive applications. The experimental results highlighted an ammonia conversion rate exceeding 99.6% and a reforming efficiency of 70.95% under the optimal conditions. Moreover, the reactor demonstrated the ability to generate 84 W of power, equivalent to a fuel cell, without requiring external heat sources. However, the formation of “hot zones” on the stainless steel (SS) reactor walls, compared to those with Cu-BN, promoted greater NH_3_ conversion by enhancing the reaction kinetics; however, these zones may also compromise the system’s long-term durability. In terms of materials, Cu-BN outperforms SS in portable configurations due to its ability to maintain uniform temperatures, reducing thermal stresses and energy losses. Furthermore, Badakhsh et al. [1] suggested that integrating this concept into microreactors could further increase efficiency and applicability, emphasizing that this system holds potential not only for ammonia decomposition but also for other hydrogen-release reactions from chemical carriers such as methanol or LOHCs (liquid organic hydrogen carriers).

### 2.3. Formic Acid Synthesis

Formic acid (FA) has emerged as a key compound in the transition towards a sustainable energy future. Its ability to act both as a hydrogen carrier and a chemical precursor in numerous processes makes it a highly valuable resource. Since its discovery in 1671, when its production was limited to natural processes, the development of modern chemistry enabled its industrial synthesis from methanol and other fossil-derived compounds. Today, the demand for formic acid continues to grow, driven not only by its energy implications but also by its role in the textile, chemical, and agricultural industries [142,143,144]. Moreover, it represents an opportunity to link carbon capture with the production of high-value-added chemicals, significantly contributing to global decarbonization goals. Its synthesis is based on multiple chemical routes, each with unique characteristics in terms of efficiency, sustainability, and economic feasibility. Traditionally, formic acid is produced through processes such as methanol oxidation and alkene hydrocarbonylation, using fossil-based feedstocks like naphtha, natural gas, coal, and heavy oils. Although these routes are highly efficient and technologically mature, their reliance on non-renewable resources limits their alignment with global sustainability goals [145,146,147].

In recent decades, alternative methods have been developed that stand out due to their potential to reduce the carbon footprint associated with HCOOH synthesis. Among these, the reduction of carbon dioxide using renewable energy or green hydrogen has emerged as one of the most promising strategies [148]. This approach not only utilizes an abundant resource like CO_2_ but also enables the direct integration of renewable energy sources, such as solar and wind, into chemical processes. Furthermore, biomass, considered a carbon-neutral source, has emerged as a viable feedstock for the sustainable production of formic acid through thermochemical or catalytic processes. This route leverages agricultural waste and residues, providing the dual benefits of waste valorization and sustainable production [144,149,150].

The efficiency and feasibility of formic acid synthesis largely depends on the catalytic systems employed. Catalysts play a fundamental role in driving reactions under milder conditions, achieving high selectivity, and minimizing energy requirements. Several studies have highlighted innovations in boron nitride-based materials, focusing on various catalytic strategies. In the electrochemical reduction of CO_2_, structural modifications of BN, such as the introduction of carbon–boron atomic groups into h-BN flakes, have shown high activity for the conversion of CO_2_ to HCOOH. This approach leverages boron’s ability to activate CO_2_, while carbon facilitates proton transfer, stabilizing reactive intermediates such as COOH* and H* [151]. In the field of photocatalysis, nanostructures like ε-Fe_2_O_3_ grafted onto h-BN have been explored for the photooxidative decomposition of formic acid under visible light, highlighting the adaptability of BN-based systems to diverse catalytic environments [152]. Additionally, Jiang et al. [153] demonstrated that h-BN is highly efficient in the photoreduction of nitrates, using formic acid as a hole scavenger, positioning BN as a sustainable non-metallic alternative. Finally, porous BN structures, such as graphene-like BN (p-BN), have also been explored for their ability to adsorb compounds like formaldehyde and serve as a support for its transformation into formic acid and methanol. These properties are driven by its high specific surface area and active functional groups on the surface, positioning p-BN as a promising material for indoor air purification and advanced environmental applications [80].

In the hydrogenation of CO_2_, BN-supported catalysts stand out due to their synergy with metallic nanoparticles, facilitating the activation of CO_2_ and hydrogen molecules. For example, boron nitride nanosheets doped with Pt have shown improved activity and selectivity for formic acid production via CO_2_ hydrogenation, according to DFT studies [154]. Another DFT study demonstrated that the homonuclear bonds in fullerene-like BN structures are particularly useful for activating and converting CO_2_ into formic acid [155]. Furthermore, donor–acceptor heterostructures combining h-BN with 2D M_2_X electron donors have shown a significant enhancement in CO_2_ activation. These structures transfer electrons to CO_2_, dramatically reducing the energy barriers in the process and achieving overpotentials as low as 0.17 V, making them highly promising platforms for sustainable catalytic applications [156]. The research conducted by Chagoya et al. [54] employing defective hexagonal boron nitride (dh-BN) as a catalyst for CO_2_ reduction was focused on its transformation into value-added molecules such as formic acid and methanol. Through a high-energy ball milling process, defects were introduced into h-BN, creating key active sites. These defects, particularly nitrogen and boron vacancies, facilitated the co-adsorption of CO_2_ and H_2_, enabling hydrogenation reactions under mild temperature and pressure conditions. The study demonstrated that dh-BN can produce methanol at temperatures as low as 20 °C and formic acid at 160 °C, with catalytic performances comparable to other heterogeneous systems. These results highlight the importance of optimizing the number of defects to maximize activity without compromising the material’s structure. Although the catalyst showed a decrease in performance after several cycles due to carbonaceous deposits (coke) on its surface, it can be regenerated via thermal treatment, underscoring its viability for sustainable applications. The study concluded that dh-BN is a promising alternative to conventional metal-based catalysts, combining efficiency, sustainability, and the ability to operate under moderate conditions. These findings open new possibilities for CO_2_ capture and reuse within the context of sustainable chemistry [54].

### 2.4. Formic Acid Decomposition

Formic acid decomposition has gained relevance in recent years as one of the most promising technologies for clean hydrogen generation, with applications extending beyond direct generation and carbon-free hydrogen storage [5,157,158]. Its versatility is further underscored by its use in tandem catalytic systems for selective hydrogenation, making it a key player in advanced industrial processes [159]. FA exhibits a gravimetric hydrogen density of 4.4%, making it an attractive energy carrier for both portable and industrial applications [9]. Moreover, its liquid nature at room temperature facilitates transport and handling compared to other hydrogen carriers [160,161]. However, its catalytic decomposition faces technical challenges, including selectivity for H_2_, catalyst durability, and efficiency under varying operational conditions [162].

The FA decomposition process follows two main pathways: (1) dehydrogenation, which produces hydrogen and carbon dioxide (HCOOH → H_2_ + CO_2_), and (2) dehydration, which generates carbon monoxide and water (HCOOH → CO + H_2_O) [163]. Selectivity toward the first pathway is crucial, as CO formation not only reduces the purity of the generated hydrogen but can also poison the electrodes in fuel cells [164]. Consequently, the design of highly selective catalysts that minimize CO formation is a central focus in this research field.

Hexagonal boron nitride materials and its derivatives have proven to be exceptional supports for catalysts employed in FA decomposition. Beyond its well-known properties, its resistance to oxidation ensures the catalytic surface’s integrity during prolonged use, making it a robust option for FA decomposition systems [19]. Advances in catalytic support design have highlighted porous BN as a transformative material. Nanotexturing BN into nanosheets or porous structures has created supports with outstanding catalytic properties. These structures provide a high surface area for metal anchoring and facilitate mass transfer [165], making it particularly effective for FA decomposition under industrial conditions.

Recent advances in catalyst design for FA decomposition have incorporated innovative strategies, including the use of bimetallic systems, support functionalization, and nanotexturing. These strategies aim to address the fundamental challenges related to selectivity, catalytic activity, and stability under demanding operational conditions. Among these approaches, the functionalization of boron nitride supports has proven particularly effective in enhancing metal–support interactions, optimizing the dispersion of metallic nanoparticles, and mitigating issues such as particle sintering or agglomeration. Although the surface of h-BN has a small amount of natural defect sites that facilitate the introduction of polar groups, functionalization strategies—both covalent and non-covalent—can improve the surface reactivity properties. These include defect derivatization, Lewis acid–base interactions, and radical covalent functionalization [46,166,167]. For instance, incorporating polar groups such as −NH_2_ and −OH into h-BN not only improves the support’s hydrophilicity, but it also provides steric stability, reducing the aggregation of metallic nanoparticles like AuPd. Furthermore, introducing impurities such as carbon into BN nanostructures, such as B_12_N_12_ nanocages, has been shown to lower activation barriers for dehydrogenation, enabling effective metal-free catalytic processes [168]. However, traditional strategies for functionalizing BN supports often involve complex, time-consuming, and energy-intensive processes that generate contaminants, potentially compromising their effectiveness [30]. Therefore, developing cleaner, more efficient functionalization methods is essential to enhance hydrophilicity, ensure high nanoparticle dispersion, and achieve superior catalytic performance for FA decomposition, balancing efficiency and sustainability in advanced support design.

Recent studies also highlight the synergistic impact of bi- and trimetallic alloys, such as Pd-Au [169], Ni-Cu [170], Pd-Cu-Cr [171], Pd-Ag [172], or Ni-Au-Pd [173]. Some of these systems combine Pd’s electronic properties with the stabilizing and electron-modifying characteristics of the second and third metals. Such innovations not only improve FA decomposition efficiency but also pave the way for developing sustainable and highly selective catalysts for CO-free hydrogen generation [173,174]. In this context, in the present review, a detailed analysis was conducted on a selection of key studies that have implemented these strategies. The functionalization with (3-aminopropyl) triethoxysilane (APTES) to produce supports modified with heteroatoms, as well as the preparation of bimetallic catalysts anchored on porous BN structures, all contributed significantly to the advancement of catalytic technology in FA-based systems. Additionally, studies that adopted a more conventional approach were included; these approaches included evaluating monometallic Pd catalysts supported on unmodified BN, allowing for a direct comparison of the influence of functionalization on catalytic performance.

Advancements in the catalytic decomposition of formic acid have led to the development of catalysts that stand out not only due to their selectivity and stability but also due to exceptional performance metrics, such as turnover frequency, activation energy, and the ability to operate efficiently at moderate temperatures. These key metrics, summarized in Table 4, are fundamental for evaluating and comparing the impact of design strategies implemented in BN supports and metal configurations. The functionalization of BN and the incorporation of bimetallic nanoparticles, such as AuPd, have proven to be key strategies for improving metal–support interactions and generating significant synergistic effects. One of the pioneering studies in this field was conducted by Guo et al. [175], who introduced an innovative catalytic system based on AuPd bimetallic nanoparticles supported on porous boron nitride nanofibers functionalized with amine groups (BNNFs-A) for the decomposition of formic acid. This design combines the high thermal and chemical stability of BNNFs-A with the uniform dispersion of metallic nanoparticles, achieved through functionalization with APTES, which promotes electronic interactions and minimizes agglomeration. Functionalization of the BNNFs with APTES successfully introduced amine groups, providing active sites for the adsorption and activation of FA molecules. Transmission electron microscopy images revealed that the AuPd nanoparticles had an average size of 2.2 nm and were uniformly distributed on the support, which was further confirmed by XRD and X-ray photoelectron spectroscopy (XPS) analyses. These studies demonstrated a strong interaction between metals and this functionalized support, highlighted by the charge transfer and a significant synergistic effect between both components.

**Scheme 2:**
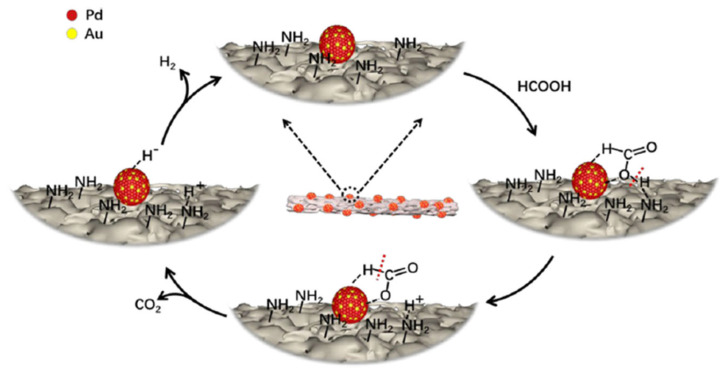
Potential mechanisms for FA dehydrogenation catalyzed by Au_1_Pd_3_/BNNFs-A. Reprinted from [175] with permission from American Chemical Society, copyright 2021.

Among the catalysts with different Au:Pd molar ratios, the Au_1_Pd_3_/BNNFs-A catalyst exhibited excellent performance, achieving complete FA decomposition at room temperature with an initial TOF of 1181.1 h^−1^, without carbon monoxide formation. This outstanding performance was attributed to the synergistic effect between gold and palladium, which optimizes the electronic structure of Pd by introducing an appropriate amount of Au, thereby enhancing catalytic activity [177]. The possible reaction mechanisms underlying this high efficiency are presented in Scheme 2, which emphasizes the importance of electronic transfer and the key steps in the catalytic dehydrogenation of FA. However, it was observed that an excess of Au reduced catalytic activity, as Au alone does not exhibit high activity for FA decomposition [178]. The analysis of the loading effect of Au_1_Pd_3_ showed that the catalytic activity reached its maximum with a metal loading of 20.5 wt%. Higher loadings caused a decline in activity due to nanoparticle aggregation on the BNNFs-A, negatively impacting dispersion and reducing the accessibility of active sites. Additionally, an increase in reaction temperature significantly improved catalytic activity, underscoring the importance of optimizing both the Au:Pd ratio and the loading and operating conditions to maximize the catalytic performance of the system. The Au_1_Pd_3_/BNNFs-A catalyst not only exhibited a low activation energy (20.1 kJ/mol) but also demonstrated remarkable stability after multiple reaction cycles. DFT calculations indicated that the amine groups in the BNNFs facilitate the initial cleavage of the O-H bond in FA molecules, while the bimetallic nanoparticles efficiently catalyze the decomposition of the formate intermediate to produce H_2_ and CO_2_ [177]. This synergistic behavior between the functionalized support and the metallic nanoparticles represents the main factor behind the high performance of the catalytic system. This system constitutes a promising advancement for sustainable hydrogen generation and serves as a model for the design of advanced catalysts in clean energy applications.

**Scheme 3:**
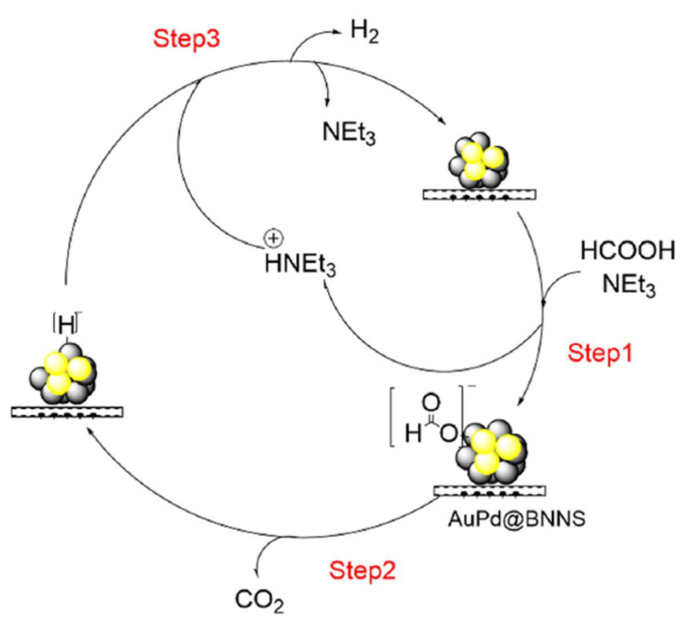
Possible reaction mechanism for H_2_ production from formic acid decomposition with Au_0.03_Pd_0.05_@BNNS catalyst. Reprinted from [72] with permission from American Chemical Society, copyright 2012.

Following a similar approach, Shaybanizadeh et al. [72] synthesized homogeneous bimetallic AuPd nanoparticles supported on boron nitride nanosheets to optimize the catalytic decomposition of formic acid for efficient hydrogen production. Catalysts were developed with varying amounts of Pd (1–5 wt%) and Au (1, 3, and 5 wt%). Among the different molar ratios evaluated, the catalyst containing 3% Au and 5% Pd (Au_0.03_Pd_0.05_@BNNS) proved to be the most effective, standing out for its performance and stability under reaction conditions. At 50 °C, this catalyst achieved an average turnover frequency of 6848 h^−1^ with 100% selectivity toward H_2_ generation, without the formation of carbon monoxide. The results indicated that the synergy between Au and Pd nanoparticles, influenced by the unique electronic properties of the system, was essential for maximizing its catalytic activity. Additionally, the BNNSs provided a highly stable and chemically inert support, preventing nanoparticle agglomeration and ensuring optimal exposure of active sites. Various parameters were evaluated to optimize the reaction, including the effect of temperature, reaction time, solvent type, catalyst loading, and different bases. Temperature was identified as a key factor: increasing it to 50 °C maximized catalytic activity, while the volume of gas produced (H_2_ + CO_2_) increased with longer reaction times. Water was identified as the optimal solvent, as it favored the dehydrogenation pathway by reducing the reaction’s energy barrier [179]. Catalyst loading also significantly impacted the reaction efficiency. An adequate loading of Au_0.03_Pd_0.05_ on the BNNSs ensured the maximum exposure of active sites, whereas excessive amounts led to nanoparticle agglomeration, reducing catalytic activity. Finally, the base N(CH_2_CH_3_)_3_, (NEt_3_), was found to be the most effective due to its ability to enhance the reaction rate. By studying these parameters, the authors proposed a plausible reaction mechanism (Scheme 3), highlighting that the use of a base like NEt_3_ facilitates the cleavage of the O-H bond in FA, generating intermediates that ultimately decompose to release CO_2_ and H_2_ [72].

**Scheme 4:**
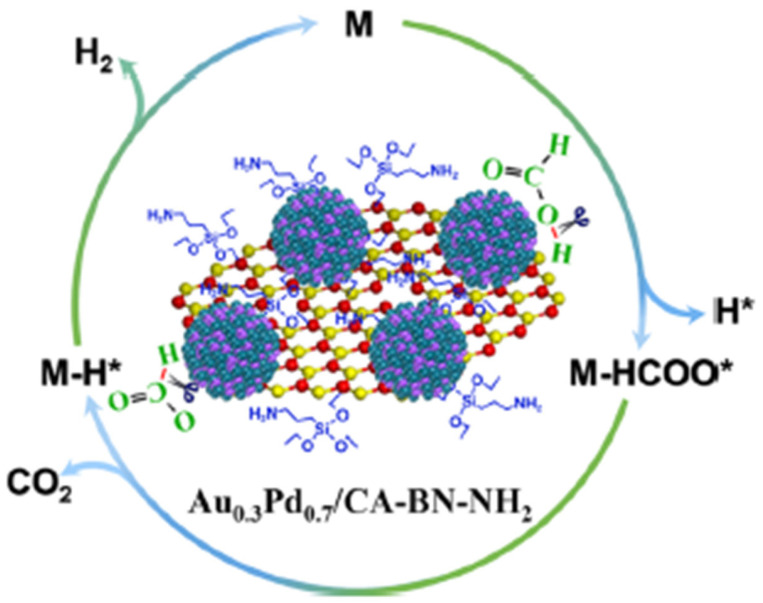
Possible reaction mechanism for H₂ production from formic acid decomposition with AuPd/CA-BN-NH₂ catalyst. Reprinted from [55] with permission from American Chemical Society, copyright 2023.

Another study conducted by Wang et al. [55] focused on the development of an innovative catalyst based on AuPd bimetallic nanoparticles supported on citric acid-modified boron nitride functionalized with amino groups (CA-BN-NH_2_). This catalytic system, referred to as Au_0.3_Pd_0.7_/CA-BN-NH_2_, demonstrated high efficiency in the decomposition of formic acid into hydrogen and carbon dioxide without the need for additional additives. Using a simple synthesis approach, citric acid was employed to create defect sites in boron nitride, facilitating the incorporation of amino groups derived from APTES. This modification endowed the support with high hydrophilicity and the ability to anchor AuPd nanoparticles uniformly, preventing their agglomeration. Structural and morphological analyses using TEM and XPS revealed nanoparticles with an average size of 3.6 nm that were homogeneously distributed on the support, which contributed to the catalyst’s high activity. The catalyst achieved 100% selectivity toward hydrogen generation and an initial turnover frequency of 7046 h^−1^, significantly surpassing other reported catalysts. This highlights how functionalized and bimetallic systems can achieve substantially higher efficiency. The combination of Au and Pd created a synergistic effect, modifying the electronic structure of Pd and optimizing critical steps in the FA dehydrogenation reaction, such as C-H bond cleavage and H₂ formation. The stability of the catalyst was evaluated over multiple cycles, showing a slight loss of activity attributed to a reduction in metal loading. However, the nanoparticle size remained constant, indicating excellent resistance to sintering. Furthermore, the effects of the Au:Pd ratio, the amounts of APTES used for functionalization, and the reaction temperatures were investigated. The results highlighted that a ratio of Au_0.3_Pd_0.7_ and 0.4 mL of APTES were optimal for maximizing the catalytic activity. Based on these findings, the authors proposed a plausible reaction mechanism for the FA dehydrogenation catalyzed by AuPd/CA-BN-NH_2_, which is illustrated in Scheme 4.

Recently, Gong et al. [76] conducted studies on catalysts based on palladium nanoparticles supported on boron nitride co-doped with carbon and oxygen (Pd/BN_C,O_). Through a high-temperature pyrolysis process, carbon and oxygen heteroatoms were introduced into the boron nitride structure, generating structural defects and increasing the density of surface functional groups, which facilitated the effective anchoring of Pd nanoparticles. Subsequently, the functionalization of the support with APTES added -NH_2_ groups to the surface. Comparative studies were conducted between functionalized catalysts (Pd/BN_C,O_-A) and non-functionalized ones (Pd/BN_C,O_). Among the non-functionalized catalysts, Pd/BN_C,O-3_ exhibited the highest TOF (95.4 h^−1^ at 298 K) and the lowest activation energy (32.9 kJ/mol), indicating that doping with carbon and oxygen atoms can reduce the activation barrier and promote FA dehydrogenation. Meanwhile, among the functionalized catalysts, Pd/BN_C,O-1_-A achieved the highest TOF (522 h^−1^ at 298 K) and, at 338 K, reached a TOF of 1560.9 h^−1^ with nearly 100% selectivity for hydrogen production and an activation energy of 23.6 kJ/mol. These results suggest that, in addition to C and O doping, the modification of the support with -NH_2_ also reduces the activation energy barrier and promotes the reaction. However, the stability of the functionalized catalyst showed a significant decline after the third cycle, whereas Pd/BN_C,O-3_ maintained its catalytic activity even after five cycles. Structural and chemical analyses revealed key differences between the functionalized and non-functionalized catalysts: Pd/BN_C,O-1_-A exhibited smaller NPs (2.1 nm compared to 2.6 nm for Pd/BN_C,O-3_) and a more uniform dispersion. However, its surface area (46.6 m^2^/g) and total pore volume (0.095 cm^3^/g) decreased by 95% and 82%, respectively, which can be attributed to the APTES functionalization and the incorporation of Pd particles, leading to pore blocking and reduced surface space. Furthermore, the XPS analysis revealed a strong metal–support interaction, attributed to electron transfer between Pd nanoparticles and functional groups on the support, enhancing FA adsorption and activation [180]. Specifically, amino groups acted as proton captors, promoting the cleavage of O-H bonds in FA molecules and improving catalytic activity. This study underscores the importance of support functionalization in the design of advanced catalysts for hydrogen production from FA, highlighting the potential of functionalized BN_C,O_ supports as a promising platform for sustainable energy applications.

While research on bimetallic systems supported on BN for FA decomposition has primarily focused on AuPd nanoparticles, a notable example is the study of CuPd nanocatalysts supported on boron nitride nanosheets, where formic acid not only acts as a hydrogen source but also as a reducing agent in environmental applications, broadening the prospects of this hydrogen carrier in advanced catalytic technologies [176]. These catalysts were synthesized using a microwave-assisted method, demonstrating high catalytic activity even with a low noble metal content. Among the catalysts studied, the Cu_8_Pd_2_/BNNS variant exhibited the best performance, with a reaction rate of 0.04044 s^−1^, significantly outperforming other similar catalysts, including those with a higher noble metal content. In this system, formic acid plays a critical role as a reducing agent. During the reaction, HCOOH is catalytically decomposed into carbon dioxide and hydrogen, the latter being responsible for reducing Cr(VI) to Cr(III). This process is facilitated by the synergistic interaction between Cu and Pd in the bimetallic alloy, as well as the metal–support interaction, where the BNNSs not only provide a stable physical support but also participate in the catalytic activation of HCOOH [181]. Comparative experiments were conducted with different Cu and Pd ratios as well as with other supports such as TiO_2_ and rGO. The results indicated that the BNNSs, due to their unique properties, are superior as a catalytic support [176].

Unlike other studies that have explored strategies such as the use of bimetallic catalysts or the functionalization of boron nitride supports to enhance catalytic efficiency, Miao et al. [75] focused exclusively on monometallic Pd catalysts supported on h-BN without additional functional modifications. This approach aimed to evaluate the inherent influence of support particle size and metal–support interactions (SMSIs) on catalytic performance for the dehydrogenation of formic acid. The study employed h-BN with three distinct particle sizes (500 nm, 1–2 µm, and 5–10 µm) as supports for Pd nanoparticles. The analyses conducted using XRD, TEM, energy-dispersive X-ray spectroscopy (EDX), and XPS revealed that supports with smaller particle sizes and higher surface areas facilitated a more uniform dispersion of Pd nanoparticles, achieving smaller particle sizes compared to larger supports. This smaller nanoparticle size demonstrated a direct correlation with higher catalytic activity, highlighting the critical role of active metal dispersion. The experimental results showed that the catalyst based on 500 nm h-BN (1-Pd/h-BN) achieved the best performance, with hydrogen production reaching 24.25 mmol H_2_/mL of FA at 160 °C and minimal CO concentrations (3.67 vol%). This performance can be attributed to the observed SMSI effects, confirmed through XPS, which identified electronic transfer from Pd to N, enhancing both stability and catalytic efficiency. Although higher temperatures favored FA dehydrogenation, a slight increase in CO formation was observed, possibly due to thermodynamic constraints. This study demonstrated that even without relying on support functionalization or bimetallic alloys, high catalytic performance can be achieved by optimizing fundamental parameters such as the support particle size and metal–support interactions. These findings underscore the potential of Pd/h-BN systems for applications in hydrogen generation from formic acid, storage, and transportation.

The catalysts studied exhibited remarkable performance in the decomposition of formic acid, but significant differences highlight the strengths and limitations of each design. Table 4 summarizes the results obtained in each of these studies. Bimetallic AuPd-based catalysts functionalized with different polar groups on BN supports, such as those studied by Guo et al. [175] and Wang et al. [55], achieved the highest turnover frequencies, followed by the catalysts developed by Shaybanizadeh et al. [73], demonstrating that functionalization and bimetallic nanoparticles are more effective for FA dehydrogenation. In comparison, monometallic Pd catalysts supported on BN, both functionalized [76] and without additional functionalization, as investigated by Miao et al. [75], offer a simpler approach, achieving lower TOFs. While these values do not reach the high activities observed in functionalized bimetallic systems, their simple design and lack of complex functionalization steps position them as viable candidates for industrial applications where robustness and ease of synthesis are critical factors.

**Figure 5:**
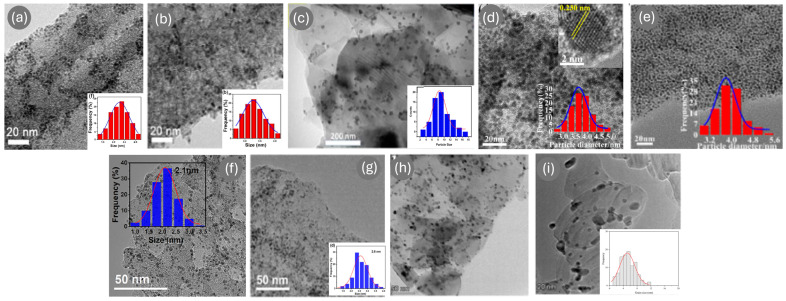
TEM images and the corresponding particle size distribution curve of the catalysts: (**a**) Au_1_Pd_3_/BNNFs-A, (**b**) Au_1_Pd_3_/BNNFs-A after 5 recycle tests, (**c**) Au_0.03_Pd_0.05_@BNNS, (**d**) Au_0.3_Pd_0.7_/CA-BN-NH_2_, (**e**) Au_0.3_Pd_0.7_/CA-BN-NH_2_ after recycle test, (**f**) Pd/BN_C,O-1_-A, (**g**) Pd/BN_C,O-1_-A after three cycles, (**h**) 1-Pd/h-BN, and (**i**) 1-Pd/h-BN after reacting for 40 min. Reprinted from [175] with permission from American Chemical Society, copyright 2021; from [72] with permission Institute of Physics (Great Britain) copyright 1990; from [55] with permission from American Chemical Society, copyright 2023; from [76] with permission from American Chemical Society, copyright 2024; and from [75] with permission from Elsevier, copyright 2024.

In Figure 5, TEM images and their corresponding particle size distributions are shown for various boron nitride-based catalysts, both functionalized and non-functionalized, to analyze their structure and behavior after recycling tests. Panels (a) and (b) display the TEM images of the Au_1_Pd_3_/BNNFs-A catalyst before and after five reaction cycles, highlighting the initially uniform distribution that maintained good dispersion with a slight increase in particle size (by 1.1 nm) after recycling. In panel (c), the boron nitride nanosheets retained their layered structure after modification with Au and Pd nanoparticles. Panels (d) and (e) correspond to the Au_0.3_Pd_0.7_/CA-BN-NH_2_ catalysts before and after five catalytic cycles, showing that the AuPd nanoparticles were highly dispersed and did not experience significant changes in size. Similarly, for the Pd/BN_C,O-1_-A catalyst before (f) and after (g) three cycles, there was no significant variation in particle size (0.5 nm), although agglomeration of Pd nanoparticles was observed, which correlates with the decline in activity after three cycles. Finally, panels (h) and (i) present the non-functionalized 1-Pd/h-BN catalyst, where the Pd nanoparticles remained morphologically stable, demonstrating the strong interaction between Pd and h-BN.

The CO formation during formic acid decomposition using BN-based systems is a critical factor in determining the final applicability of these materials. In this review, we comparatively analyzed the published data on metallic nanoparticles supported on porous boron nitride. Notably, the majority of the consulted references [55,72,76,175] reported that complete formic acid decomposition into hydrogen and CO₂ occurs without CO formation. This experimental evidence supports the effectiveness of BN-supported catalysts in achieving CO-free formic acid dehydrogenation. In contrast, the only study reporting CO formation during formic acid dehydrogenation involved a series of Pd/h-BN materials, with varying BN particle sizes (reference [75]). Although the reported CO concentration was relatively low (3.67% vol.), the fact that the CO yield increased with the formic acid decomposition temperature suggests a limitation of these catalysts for applications such as hydrogen storage, transportation, or production of an in situ supply.

## 3. Conclusions

In the processes of ammonia synthesis and decomposition, the use of boron nitride (BN) as a support for metallic catalysts enables the addition of promoters (e.g., BaO or Cs_2_O), which interact more specifically with the active phases (Ru and Ni). Interestingly, employing these supports facilitates the formation of optimal catalytic centers for ammonia-related reactions, namely the centers consisting of ensembles of metal atoms in a B5-type surface structure.

The versatility of these supports is also evident in the synthesis and decomposition of formic acid (FA). In the synthesis of FA, it is worth highlighting the ability of these BN materials to produce this valuable reactant and a potential hydrogen source by consuming CO_2_ in the presence of strong bases. Regarding the formic acid decomposition reaction, BN supports facilitate the incorporation of metallic nanoparticles along with functionalized surfaces containing basic species, such as amine groups, which enhance the catalytic performance in the FA dehydrogenation reaction.

In general, since BN supports consist of non-oxide materials, they provide a surface chemistry that enables the tailor-made design—through functionalization—of suitable catalytic sites for each type of reaction. Finally, before the industrial applications of these new catalytic materials, it is compulsory to evaluate the aspects related to their cost scalability issues. Also, it should be noted that the large-scale synthesis of high-purity BN supports or their functionalization processes, when using non-ecofriendly reactants, may lead to unexpected environmental impacts.

## Figures and Tables

**Table 2:** Summary of reported H_2_ production rates for ruthenium-based catalysts in ammonia decomposition.

Ref.	Catalyst	GHSV (ml/g_cat_·h)	wt%	T (°C)	H_2_ Production Rate (mmol/g_cat_·s)
[114]	Ru/h-BN	-	1	450	0.32
[132]	Nano-Ru@SiO_2_	30,000	-	450	0.38
[130]	Ru/AC	30,000	4.80	450	0.02
[129]	Ru/Al_2_O_3_	150,000	5.00	450	0.13
[130]	K-Ru/CNNTs	30,000	4.80	450	0.54
[136]	K-Ru/MgO-CNTs	60,000	4.85	450	0.94
[127]	Ru/MgO	60,000	4.70	400	0.07
[126]	K-Ru/MgO	30,000	4.60	400	0.06

**Table 3:** Summary of reported H_2_ production rates for nickel-based catalysts in ammonia decomposition.

Ref.	Catalyst	GHSV (ml/g_cat_·h)	wt%	T (°C)	H_2_ Rate (mmol/g_cat_·s)
[137]	Ni/CeO₂-BN	30,000	9.5	600	0.49
[132]	Ni@SiO_2_	30,000	-	500	0.20
[131]	Ni/AC	-	5	500	-
[139]	Ni/Al_2_O_3_	30,000	-	450	0.08
[128]	Ni/Al_2_O_3_	-	8.9	500	1.02
[128]	Ni/Al-Ce_0.8_Zr_0.2_O_2_	-	8	500	2.20
[128]	Ni/Ce_0.8_Zr_0.2_O_2_	-	10.7		1.80
[129]	Ni/CNTs	30,000	-	500	0.05
[140]	Ni/SiO_2_	-	10	600	0.19
[140]	Ni/SiO_2_/ Al_2_O_3_	-	65	600	0.41

**Table 4:** Summary of some reported metrics for the performance of boron nitride-based catalysts in FA decomposition.

Ref.	Catalyst	Ea (kJ/mol)	T (°C)	TOF (h^−1^)	NP Size (nm)
[175]	Au_1_Pd_3_/BNNFs-A	20.1	25	1181	2.2
[72]	Au_0.03_Pd_0.05_@BNNSs	-	50	6848	8.4
[55]	Au_0.3_Pd_0.7_/CA-BN-NH_2_	29.7	20	7046	3.6
[76]	Pd/BN_C,O-1_-A	23.6	65	1561	2.1
[176]	Cu_8_Pd_2_/BNNSs	26.07	25	-	3.4
[76]	1-Pd/h-BN	-	160	913	6
